# Adherence to Nutrition and Physical Activity Cancer Prevention Guidelines and Development of Colorectal Adenoma

**DOI:** 10.3390/nu10081098

**Published:** 2018-08-16

**Authors:** Lindsay N. Kohler, Robin B. Harris, Eyal Oren, Denise J. Roe, Peter Lance, Elizabeth T. Jacobs

**Affiliations:** 1 Department of Health Promotion Sciences, Mel and Enid Zuckerman College of Public Health, University of Arizona, Tucson, AZ 85724, USA; 2 Department of Epidemiology and Biostatistics, Mel and Enid Zuckerman College of Public Health, University of Arizona, Tucson, AZ 85724, USA; rbharris@email.arizona.edu (R.B.H.); eoren@sdsu.edu (E.O.); droe@email.arizona.edu (D.J.R.); jacobse@email.arizona.edu (E.T.J.); 3 Department of Medicine, University of Arizona Cancer Center, Tucson, AZ 85724, USA; plance@azcc.arizona.edu; 4 Division of Epidemiology and Biostatistics, San Diego State University School of Public Health, San Diego, CA 92182, USA

**Keywords:** adherence, colorectal adenoma, cancer prevention guidelines, diet, physical activity

## Abstract

Adherence to the American Cancer Society’s (ACS) Nutrition and Physical Activity Cancer Prevention Guidelines is associated with reductions in overall cancer incidence and mortality, including site-specific cancers such as colorectal cancer. We examined the relationship between baseline adherence to the ACS guidelines and (1) baseline adenoma characteristics and (2) odds of recurrent colorectal adenomas over 3 years of follow-up. Cross-sectional and prospective analyses with a pooled sample of participants from the Wheat Bran Fiber (*n* = 503) and Ursodeoxycholic Acid (*n* = 854) trials were performed. A cumulative adherence score was constructed using baseline self-reported data regarding body size, diet, physical activity and alcohol consumption. Multivariable logistic regression demonstrated significantly reduced odds of having three or more adenomas at baseline for moderately adherent (odds ratio [OR] = 0.67, 95% confidence intervals [CI]: 0.46–0.99) and highly adherent (OR = 0.50, 95% CI: 0.31–0.81) participants compared to low adherers (*p*-trend = 0.005). Conversely, guideline adherence was not associated with development of recurrent colorectal adenoma (moderate adherence OR = 1.16, 95% CI: 0.85–1.59, high adherence OR = 1.23, 95% CI: 0.85–1.79).

## 1. Introduction

Colorectal cancer (CRC) is the third most commonly diagnosed cancer and the second leading cause of cancer mortality among men and women in the United States [1]. Despite decreasing incidence rates for colorectal cancer over the past two decades in the United States among men and women aged 50 years and older, the American Cancer Society (ACS) estimates that there will have been 95,520 new cases of colon cancer and 39,910 new cases of rectal cancer diagnosed in 2017, with 49,190 deaths from these malignancies [2]. Increased screening rates among those aged 50 years and older have contributed to the reduction in colorectal cancer rates over the past 20 years in this age group through early detection and removal of adenomatous polyps, the precursors to colorectal cancer [3,4]. However, over the same time period there has been a progressive increase in colorectal cancer incidence in individuals without a known genetic predisposition to the disease in individuals under the age of 50 years [5].

Approximately 96% of colorectal cancers are adenocarcinomas, which develop in colorectal epithelial cells [6]. Adenomas, the most common type of colorectal polyp, are benign neoplasms that up to 50% of all individuals will develop in their lifetime [7,8]. However, adenomas typically cause no symptoms and a minority may progress asymptomatically to cancer unless removed. The United States Preventive Services Task Force (USPSTF) recommends that adults aged 50 to 75 years should be screened for colorectal cancer every 1–10 years depending on the specific screening test and personal risk factors. However, it is currently estimated that only half of those recommended for colorectal screening are following these guidelines [9]. In order to prevent CRC in those unable or unwilling to undergo the current colorectal screening procedures, further strategies for preventing colorectal neoplasia are essential.

There are several innate factors that may increase the risk of colorectal cancer [9] but there are also several modifiable risk factors for CRC such as physical inactivity, overweight and obesity [10]. Overconsumption of energy-rich foods, high consumption of red and/or processed meat, deficiency in some micronutrients or phytochemicals, moderate to heavy alcohol consumption and smoking early in life [10] have been shown to increase the risk for CRC [10]. 

Nutrition and physical activity guidelines for cancer prevention have been designed by the United States Department of Health and Human Services along with leading cancer organizations in order to provide recommendations for addressing these factors [11,12]. Our recent systematic review of 12 large cohort studies [13] found that participants following behavior-associated cancer prevention guidelines for modifiable factors, such as body weight, physical activity, diet and alcohol consumption, had a reduced risk of cancer incidence, cancer mortality and all-cause mortality [11,12]. However, to date, the association between following American Cancer Society (ACS) or other healthy-lifestyle recommendations and odds for the development of colorectal cancer precursor lesions has not been addressed. The present study assesses the relationship between adherence to the ACS nutrition and physical activity cancer prevention guidelines and (1) baseline adenoma characteristics (histology, size, multiplicity) and (2) new (recurrent) colorectal adenoma occurrence.

## 2. Materials and Methods 

### 2.1. Study Population

We pooled data from two randomized, controlled, double blind, Phase III clinical trials conducted at the University of Arizona Cancer Center (UACC). The effect of either wheat bran fiber (WBF) [14] or ursodeoxycholic acid (UDCA) [15] was evaluated against the development of a recurrent colorectal adenoma. The present analyses were conducted for 1357 participants in the pooled sample with complete data for baseline adenoma characteristics, diet and physical activity measures and follow-up for evaluation of recurrent colorectal adenomas. The University of Arizona Human Subjects Protection Program previously approved both studies. Each participant provided written informed consent prior to trial enrollment.

Recruitment and Data Collection: Participants were originally recruited from Phoenix and Tucson gastroenterology practices from 1990–1995 for WBF and 1995–1999 for UDCA. Inclusion criteria included men and women aged 40 to 80 years who had at least one adenoma (≥3 mm) removed via a colonoscopy within 6-months prior to study enrollment. Mean time from trial randomization to follow-up colonoscopy was 3.1 and 3.2 years for the WBF and UDCA trials, respectively [14,15]. WBF trial participants were randomized to a daily wheat bran fiber supplement (13.5 g/day) or a low-fiber supplement (2.0 g/day); UDCA trial participants were randomized to receive 8–10 mg UDCA per kilogram of body weight or placebo [15]. Primary findings demonstrated that neither the WBF supplement nor the UDCA treatment reduced the number of recurrent colorectal adenomas [15,16]. 

### 2.2. Outcome Ascertainment

Medical records and pathology reports were used to collect baseline and recurrent adenoma characteristics such as number, size, location and histology [14,15]. Presence of a recurrent colorectal adenoma was defined as yes or no. Advanced colorectal adenoma is defined as an adenoma >1 cm in size or an adenoma of any size with tubulovillous or villous histology. Advanced adenomas are those at greatest risk for progression to colorectal cancer. Metachronous adenoma risk, that is, the development of recurrent adenoma following adenoma resection (polypectomy), is increased in individuals with baseline advanced adenoma compared to those with non-advanced adenoma and metachronous (recurrent) adenoma risk is also increased in individuals with >2 non-advanced baseline adenoma [17,18]. 

### 2.3. Nutrition and Physical Activity Cancer Prevention Guidelines Score

An *a priori* adherence score was constructed, based upon previous work [13], for adherence to the 2012 ACS cancer prevention guidelines for nutrition and physical activity [12]. The guidelines focused on an overall pattern of lifestyle behaviors that included body weight, physical activity, diet and alcohol consumption. Frequency questionnaires were used to collect baseline diet and physical activity data from 1990–1992 for WBF and 1995–1996 for UDCA. Diet was assessed utilizing the Arizona Food Frequency Questionnaire (AFFQ), which is a semi-quantitative, 175-item validated questionnaire that queries participants to report how often and how much they consumed each food item over the past 12-month period [19]. Physical activity was assessed utilizing the Arizona Activity Frequency Questionnaire (AAFQ), a 59-item, validated questionnaire that asks participants about usual physical activity in the past four weeks [20]. 

Adherence scores were based upon equally weighted ACS recommendations on a 0–2 point scale (not meeting the recommendation at all = 0 points, partially meeting the recommendation = 1 point, fully meeting the recommendation = 2 points). The overall score, summed from individual recommendations, ranged from not adherent at all to the recommendations (0 points) to fully adherent to all four lifestyle factor recommendations (8 points). Adherence scores were categorized into low (0–2 points), moderate (3–5 points) and high (6–8 points). Recommendations for each lifestyle factor, how they were measured, how scores were assigned based upon the guidelines and the proportion of the study sample within each adherence score category are shown in Table 1. Although the ACS guidelines recommend choosing whole grains over refined grains, the proportion of whole grain consumption was not included in the adherence score for these analyses because (1) grains-related questions in the food frequency questionnaire were vague in distinguishing whole versus refined grains and (2) the food frequency questionnaire was updated between the WBF and UDCA trials, which could have led to misclassification. Smoking status was not included in the adherence score but was included as a potential confounder in the current analyses.

Maintaining a healthy body weight was scored based upon body mass index (BMI, in kg/m^2^) from height and weight reported at baseline. Fully meeting the recommendation (2 points) was given to those with a BMI within normal range (18.5–25 kg/m^2^). Not meeting the recommendation at all (0 points) was given to those with a BMI in the obese category (>30.0 kg/m^2^). One point was given to those partially meeting the recommendation with a BMI in the overweight range (25–30 kg/m^2^). Underweight participants (<18.5 kg/m^2^) were excluded from the present analysis. 

Adopting a physically active lifestyle was evaluated by MET [21] scores from the AFFQ recreational activities section. The minimum standard of 30 min on 5 days (2.5 h/week) of moderate activity (3.5 METs) is equal to 8.75 MET-hours per week. Any participant performing less than 8.75 MET-hours per week received a score of zero points for not meeting the recommendation at all. One hour per day, 5 days a week (5.0 h/week), of moderate activity (3.5 METs) is equal to 17.5 MET-hours/week. Therefore, 8.75 to 17.5 MET-hours/week was considered partially meeting the recommendation and received 1 point. Meeting “preferable” levels of greater than 17.5 MET-hours/week received 2 points for fully meeting the recommendation. 

Consumption of a healthy diet with an emphasis on plant sources was assessed with three sub-scores that were constructed and summed to capture the recommended dietary pattern. For the first diet sub-score, 1 point was assigned for meeting the recommended number of 5 servings of fruits and vegetables each day. The number of servings was measured from the following food group categories: Fruits, Fruit Juice, Vegetables and Vegetable Juice. One or 2 points were assigned for diet quality based upon being in the 2nd or 3rd sex-specific tertile of total carotenoids, respectively, which included beta carotene, alpha carotene, beta cryptoxanthin, lycopene and lutein plus zeaxanthin combined. Limiting the consumption of processed and red meats was assessed with a sex-specific quartile distribution with the lowest quartile receiving 3 points and the highest quartile receiving zero points. The diet sub-scores were summed for a potential total of 6 points. Dietary pattern scores were further collapsed into 0 points for those with 0–1 summed diet scores, 1 point for those with 2–4 summed diet scores and 2 points for those with 5–6 summed diet scores. 

Alcohol consumption was captured in the AFFQ in terms of total grams of alcohol per day. One drink was estimated as 14 g of alcohol or approximately a 12 ounce regular beer, 5 ounce glass of wine, or 1.5 ounce shot of 80-proof distilled spirit [22]. Nondrinkers were assigned 2 points, moderate drinkers consuming the limit or less (1 drink per day for women or 2 drinks per day for men) were assigned 1 point and heavy drinkers consuming more than the limit were assigned zero points. 

### 2.4. Statistical Analysis

Descriptive statistics were generated for outcome variables, exposure variables and demographic variables. Chi-square tests were used to test associations of the chosen variables for participants with and without adenoma recurrence and for recurrent subjects stratified by sex. Current literature suggests potential confounders include age, previous polyps, family history of colorectal adenomas and/or cancer and aspirin use [23,24,25]. Additional covariates were examined and included if the measure of association changed by at least 10% when entered in the model [26]. Multiple logistic regression models were utilized to assess the association of adherence score with adenoma recurrence and to evaluate potential interaction between adherence score and (1) sex as a biological variable (2) study and (3) smoking. Statistical significance was determined at an α level of 0.05 utilizing two-sided tests. Data from the trials were merged and managed using Stata version 14.1 software (StataCorp LP, College Station, TX, USA).

## 3. Results

Table 1 demonstrates high adherence to the guidelines was achieved by 19.1% of the sample population while 12.2% and 68.7% attained low and moderate adherence, respectively. Baseline characteristics by category of adherence score are shown in Table 2. In general, the proportion of those in the highest versus the lowest categories of adherence did not vary by age or race, though there was a greater percentage of college graduates among those in the high adherence group (35.4%) compared to the low adherence group (29.4%). Highly adherent participants were also more likely to have a lower BMI and to perform more physical activity than those in the low adherence category. 

Table 3 presents the adjusted odds ratios for the association between adherence score categories and baseline (prevalent) colorectal adenoma characteristics from multivariate logistic regression models. In the pooled sample, reduced odds of having three or more prevalent adenomas at baseline were shown for moderately adherent (OR = 0.67, 95% CI: 0.46–0.99) and highly adherent (OR = 0.50, 95% CI: 0.31–0.81) participants compared to those with low adherence (*p*-trend = 0.005). No statistically significant associations were shown between guideline adherence and baseline (prevalent) adenoma size or villous histology in the pooled sample. No heterogeneity of effect was demonstrated between sexes for the relationship between adherence score category and any of the baseline (prevalent) adenoma characteristics. 

Table 4 presents the association between metachronous (recurrent) colorectal adenoma and adherence score category from multivariate logistic regression models. In the pooled sample, there were no statistically significant associations between guideline adherence and development of a new adenoma upon follow-up. The odds of having a recurrent colorectal adenoma were 1.16 times (95% CI: 0.85–1.59) greater for those who were moderately adherent to the guidelines and 1.23 times greater (95% CI: 0.85–1.79) for those individuals who had high adherence compared to those with low adherence but this finding was not statistically significant. No significant interactions were observed by sex.

## 4. Discussion

The results of the present study demonstrate that adherence to the cancer prevention guidelines was associated with lower odds of >2 adenomas at baseline. Those who were more adherent to the guidelines were significantly less likely to have >2 adenomas, which is an established marker of increased risk for recurrent adenoma and colorectal cancer [27]. In contrast, there were no statistically significant associations observed for guideline adherence and odds of recurrent adenoma surveillance colonoscopy 3–5 years later. 

To our knowledge, no studies have evaluated the association between ACS guideline adherence specifically and the risk for precancerous colorectal lesions. However, prior studies have examined similar indices that include the variables employed in the ACS guidelines [28,29,30]. In a recent report by Knudsen et al. [30], the relationship between a lifestyle score and detection of high-risk adenomas, including multiple lesions, was assessed among more than 6000 Norwegians undergoing either fecal immunochemical testing or flexible sigmoidoscopy screening [30]. They observed a statistically significant inverse relationship between number of healthy lifestyle factors, including lower BMI, greater physical activity and higher fruit and vegetable consumption and odds for the development of advanced colorectal neoplasia [30]. 

In a case-control study with 1444 cases and 3764 controls by Fu et al., researchers created a scale of lifestyle factors such as higher BMI, non-use of NSAIDS, smoking, obesity and low fiber and calcium intake and found that the more adverse lifestyle factors present, the higher the risk of both adenomatous and hyperplastic polyps, with similarly increased odds observed for both non-advanced and advanced lesions, including multiple polyps [29]. Finally, in another case-control study by Tabung et al. [28], a healthy lifestyle index was created among 143 participants undergoing colonoscopy. The index included smoking habits, alcohol use, physical activity, BMI and intake of fat and fruits and vegetables [28]. Overall, they observed no association between number of healthy behaviors and odds for colorectal neoplasia. However, they found that for participants who reported no use of NSAIDs, those in the healthy lifestyle category had 72% lower odds of any colorectal adenoma as compared to those in the unhealthy category (OR 0.28; 95% CI 0.08, 0.98) [28]. In addition, a one-unit increase in the lifestyle index significantly reduced odds of any adenoma by 53% (OR 0.47; 95% CI 0.26, 0.88) [28]. Taken together, the results of prior studies as well as the present work suggest that adherence to healthier lifestyle behaviors may have a role in prevention of incident colorectal lesions, particularly multiple lesions. However, after detection and removal of adenomas, our work suggests that adherence to the ACS guidelines has no impact on recurrent adenoma rate 3–5 years later. 

Another factor which warrants further consideration is that although a statistically significant association between the adherence score and odds of a recurrent adenoma after polypectomy was not observed in the present study, recent work has demonstrated a significant reduction in colorectal cancer risk (27–52%) for those who highly adhere to the ACS guidelines versus those with low adherence [13,31]. It is possible that the 3–5 year window between baseline and follow-up colonoscopies among participants in the current work is not long enough to detect an impact of the cancer prevention guidelines. It may also be that the impact of a healthier lifestyle occurs at a later stage of the carcinogenesis pathway, rather than in the initiation of a new lesion after colonoscopic removal. Another possible factor is that our study population’s median baseline age was >60 years. Perhaps by this age range, epigenetic and other changes related to adverse lifestyle may no longer be reversible.

The major strengths of the current study include availability of data from a prospective cohort of over 1300 participants with complete data on a wide range of available baseline nutrition, physical activity and recurrent colorectal adenoma outcome data. This study is not without limitations. Self-reported diet and physical activity are susceptible to measurement error or misclassification bias. In addition, the lifestyle pattern at the time of screening may not reflect participants’ health behaviors leading up to the onset of their adenoma development. Various behaviors included in the ACS adherence score may also cluster causing potential challenges in parsing out the contributing components. Further, the ACS recommendation “maintenance of a healthy weight throughout life” could not be precisely assessed because height and weight data were only available at study baseline rather than for younger ages. Finally, we were unable to adjust for the number of colonoscopies that occurred prior to study entry and it is possible that those who were more adherent to the guidelines were more likely to have regular screening colonoscopies and therefore less likely to have multiple adenomas discovered at the baseline colonoscopy. 

## 5. Conclusions

In summary, these results suggest that following an overall pattern of healthy behaviors as recommended in the ACS Nutrition and Physical Activity Cancer Prevention guidelines is associated with a reduction in odds for multiple (>2) non-advanced colorectal adenomas at baseline colonoscopy. However, no association with adhering to the guidelines and odds of developing a recurrent colorectal adenoma was observed over the 3–5-year follow-up period in our sample population. This is an important area for further research as the presence of multiple adenomas increases the risk of development of CRC. Prevention of multiple adenomas may have an impact on colonoscopy screening rates as well, as multiplicity is an indicator for more frequent surveillance. 

## Figures and Tables

**Table 1:** Components of the guideline adherence score and distribution in the study sample.

	Score	Description	All *n* (%)
Overall adherence score	Low	0–2 points	204 (12.2)
	Moderate	3–5 points	1147 (68.7)
	High	6–8 points	319 (19.1)
Adherence score component			
Body mass index (BMI)	0	>30 kg/m^2^	476 (28.5)
	1	>25–≤30 kg/m^2^	749 (44.9)
	2	18.5–≤25 kg/m^2^	445 (26.7)
Physical Activity (PA)	0	<8.75 MET h/week	658 (39.4)
	1	8.75–17.5 MET h/week	421 (25.2)
	2	>17.5 MET h/week	591 (35.4)
Overall Diet ^1^	0	0–1 sub-score sum	209 (12.5)
	1	2–4 sub-score sum	947 (56.7)
	2	5–6 sub-score sum	514 (30.8)
Fruit & Vegetables sub-score	0	<5 servings/day fruits plus veg	836 (50.1)
	1	≥5 servings/day fruits plus veg	834 (49.9)
Quality sub-score	0	1st tertile of total carotenoids	540 (32.3)
	1	2nd tertile of total carotenoids	564 (33.8)
	2	3rd tertile of total carotenoids	566 (33.9)
Red & processed meat sub-score	0	Highest quartile	421 (25.2)
	1		423 (25.3)
	2		420 (25.2)
	3	Lowest quartile	406 (24.3)
Alcohol	0	Men ≥3, Women ≥2 drinks/day	153 (9.2)
	1	Men 1–2, Women 1 drink/day	921 (55.2)
	2	Non-drinker	596 (35.7)

^1^ Overall Diet score is generated from the summation of the fruit & vegetable, quality and red & processed meat sub-scores then collapsed into 3 categories (0–1, 2–4, 5–6) for subsequent overall diet adherence scores (0, 1, 2).

**Table 2:** Baseline characteristics of participants in the pooled sample, by categories of adherence ^1^.

	Adherence Score Category (Points)		
	0–2	3–5	6–8
*n* (%)	204 (12.2)	1147 (68.7)	319 (19.1)
Age, years ^2^	62.8 ± 8.4	65.8 ± 8.6	68.0 ± 8.1 *
White, *n* (%)	195 (95.6)	1085 (94.6)	303 (95.0)
College graduate, *n* (%)	60 (29.4)	409 (35.7)	113 (35.4)
BMI, kg/m^2^	32.5 ± 4.4	28.2 ± 4.3	24.5 ± 2.7 *
Physical activity, MET-h/week	4.1 ± 5.0	16.7 ± 29.2	31.3 ± 32.2 *
Diet			
Total energy, kcal/day	2135 ± 797	1955 ± 757	1946 ± 726 *
Fruit and veg, servings/day	5.0 ± 3.3	5.7 ± 3.7	6.9 ± 3.7 *
Total carotenoids, mg/day	13.4 ± 9.1	13.8 ± 8.7	15.1 ± 7.4 *
Red and processed meat, servings/day	2.0 ± 1.0	1.4 ± 0.8	1.1 ± 0.7 *
Alcohol			
Nondrinker at baseline, *n* (%)	22 (10.8)	391 (34.1)	183 (57.4) *
Intake among drinkers, drinks/day	1.3 ± 1.7	0.8 ± 1.2	0.7 ± 0.7 *
Current smoker, *n* (%)	34 (16.7)	137 (11.9)	38 (11.9)
Family history CRC, *n* (%)	51 (25.0)	267 (23.3)	66 (20.7)
Previous polyps, *n* (%)	78 (38.2)	489 (42.6)	131 (41.1)
Aspirin use in last 4 weeks, *n* (%)	49 (24.0)	340 (29.6)	113 (35.4) *
Number of colonoscopies during study period	1.8 ± 0.8	1.8 ± 0.8	1.8 ± 0.9
Baseline adenoma characteristics			
Multiplicity, ≥3 adenomas, *n* (%)	42 (15.0)	192 (68.3)	47 (16.7)
Large size, >1cm, *n* (%)	89 (13.1)	463 (67.9)	130 (19.1)
Tubulovillous/villous histology, *n* (%)	49 (14.1)	228 (65.5)	71 (20.4)
Proximal location, *n* (%)	109 (12.8)	594 (69.6)	150 (17.6)

^1^ Some percentages do not add up to 100% because of missing data or rounding. BMI, body mass index; MET-h/week, metabolic equivalent hours per week; CRC, colorectal cancer. ^2^ Mean ± SD (all such values). * *p*-value < 0.05. Non-parametric test for trend for continuous variables and ANOVA for categorical variables.

**Table 3:** Adjusted ORs (95% CI) for the association between category of guideline adherence and baseline colorectal adenoma characteristics for pooled sample and by sex and study.

	Baseline Adenoma Characteristics (OR, 95% CI) ^1^					
Acs Adherence Score Category	Multiplicity (≥3 Adenoma)		Large Size (≥1 cm)		Villous Histology	
	*n* (%)	OR (95% CI)	*n* (%)	OR (95% CI)	*n* (%)	OR (95% CI)
Pooled sample						
Low (0–2)	204 (12.2)	1.00	89 (13.1)	1.00	49 (14.1)	1.00
Moderate (3–5)	1147 (68.7)	0.67 (0.46–0.99)	463 (67.9)	0.85 (0.63–1.15)	228 (65.5)	0.78 (0.55–1.11)
High (6–8)	319 (19.1)	0.50 (0.31–0.81)	130 (19.1)	0.85 (0.59–1.22)	71 (20.4)	0.89 (0.58–1.36)
*p*-Trend		0.005		0.455		0.765
Men						
Low (0–2)	141 (12.3)	1.00	57 (12.3)	1.00	34 (14.5)	1.00
Moderate (3–5)	791 (68.7)	0.62 (0.40–0.97)	319 (69.1)	0.92 (0.64–1.34)	153 (65.4)	0.73 (0.47–1.12)
High (6–8)	219 (19.0)	0.47 (0.27–0.82)	86 (18.6)	0.83 (0.53–1.30)	47 (20.1)	0.78 (0.46–1.30)
*p*-Trend		0.011		0.405		0.443
Women						
Low (0–2)	63 (12.1)	1.00	32 (14.6)	1.00	15 (13.2)	1.00
Moderate (3–5)	356 (68.6)	0.82 (0.38–1.79)	144 (65.5)	0.68 (0.40–1.17)	75 (65.8)	0.90 (0.48–1.70)
High (6–8)	100 (19.3)	0.56 (0.21–1.48)	44 (20.0)	0.85 (0.45–1.60)	24 (21.1)	1.16 (0.55–2.45)
*p*-Trend		0.221		0.809		0.577
*p*-Interaction ^2^		0.8360		0.4363		0.8597

^1^ ORs adjusted for age, sex (except for stratified analysis) and study (except for stratified analysis), ^2^ *p* for interaction calculated by likelihood ratio test.

**Table 4:** Adjusted ORs (95% CIs) for the association between category of adherence and recurrent colorectal adenoma occurrence for pooled sample and by sex and study.

				Recurrent Adenoma Occurrence (OR, 95% CI) ^1^					
Adherence Score Category	Any Recurrent Adenoma			Multiplicity (≥3 Adenoma)		Large Size (≥1 cm)		Villous Histology	
	*n* (%)		OR (95% CI)	*n* (%)	OR (95% CI)	*n* (%)	OR (95% CI)	*n* (%)	OR (95% CI)
Pooled sample									
Low (0–2)	105 (13.8)		1.00	22 (11.2)	1.00	14 (8.4)	1.00	11 (9.2)	1.00
Moderate (3–5)	504 (66.2)		1.16 (0.85–1.59)	130 (66.0)	0.97 (0.58–1.59)	112 (67.1)	1.40 (0.78–2.51)	88 (73.3)	1.35 (0.70–2.58)
High (6–8)	152 (20.0)		1.23 (0.85–1.79)	45 (22.8)	1.11 (0.62–1.98)	41 (24.6)	1.83 (0.95–3.51)	21 (17.5)	1.08 (0.50–2.33)
*p*-Trend			0.294		0.611		0.055		0.938
Men									
Low (0–2)	67 (11.9)		1.00	18 (11.6)	1.00	12 (9.6)	1.00	10 (11.1)	1.00
Moderate (3–5)	385 (38.4)		1.00 (0.69–1.45)	98 (63.2)	0.85 (0.48–1.49)	85 (68.0)	1.12 (0.59–2.15)	65 (72.2)	1.00 (0.50–2.02)
High (6–8)	111 (19.7)		1.00 (0.64–1.56)	39 (25.2)	1.10 (0.58–2.12)	28 (22.4)	1.27 (0.61–2.67)	15 (16.7)	0.76 (0.32–1.77)
*p*-Trend			0.983		0.531		0.494		0.435
Women									
Low (0–2)	17 (8.6)		1.00	4 (9.5)	1.00	2 (4.8)	1.00	1 (3.3)	1.00
Moderate (3–5)	138 (69.7)		1.69 (0.92–3.09)	32 (76.2)	1.30 (0.43–3.89)	27 (64.3)	2.45 (0.56–10.64)	23 (76.7)	4.42 (0.58–33.38)
High (6–8)	43 (21.7)		2.02 (1.01–4.06)	6 (14.3)	0.92 (0.25–3.40)	13 (31.0)	4.58 (0.98–21.39)	6 (20.0)	4.15 (0.48–35.56)
*p*-Trend			0.061		0.750		0.021		0.306
*p*-Interaction ^2^			0.2152		0.3877		0.3253		0.1281

^1^ ORs adjusted for age, study (except for stratified analysis), baseline multiplicity (except villous histology analyses) and sex (except for stratified analysis). ^2^ *p* for interaction calculated by likelihood ratio test.
